# A Rare Presentation of Extraosseous Ewing Sarcoma Manifesting as a Dumbbell Tumor on the Nape of the Neck

**DOI:** 10.1155/2022/5451319

**Published:** 2022-09-19

**Authors:** Abrar A. AlAtwan, Mousa Behbehani, Ali Sayed Ali

**Affiliations:** Mubarak AlKabeer Hospital, Jabriya, Hawalli, Kuwait

## Abstract

Ewing sarcoma is a rare poorly differentiated and highly malignant tumor primarily affecting the skeletal system. It most commonly presents during the first two decades of life and rarely might it be of extraskeletal origin. Majority of extraskeletal cases are reported in the paravertebral and lower limb, with very few cases reported in the cervical spine region. We report a case of extraskeletal Ewing sarcoma in a 42-year-old man who presented with a 1-year history of neck swelling associated with neck pain, diagnosed by computed tomography and magnetic resonance imaging scans, in conjunction with histological analysis. Very few cases of cervical EES presenting as dumbbell tumors have been documented in the literature especially in this age group.

## 1. Introduction

Ewing sarcoma (ES) was first described in 1921 by James Ewing as an undifferentiated small round cell tumor involving the diaphysis of long bones which regressed with radiation treatment, in contrast to osteosarcomas [1]. Extraosseous or extraskeletal Ewing sarcoma (EES) was first mentioned in literature by Tefft et al. [2] in 1969, which was further explored in detail by Angervall and Enzinger [3] in 1975. Around 400 cases of Ewing sarcoma family of tumors (ESFT) are diagnosed annually in the United States [4, 5], and 20-30% of them are EES [6]. EES is a rare sarcoma of neuroectodermal origin of the ESFT which can present anywhere in the body from head to toe. It commonly arises in the trunk, lower extremities, chest wall, and retroperitoneum [3, 4]. EES tends to present more commonly in adult populations when compared to Ewing sarcoma which rarely presents past the third decade of life [4, 6].

## 2. Case Presentation

**Figure 1:**
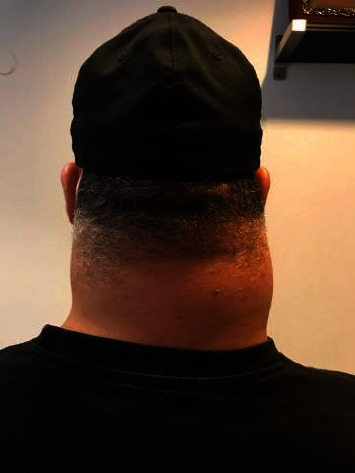
Large bilateral nape of neck swelling.

**Figure 2:**
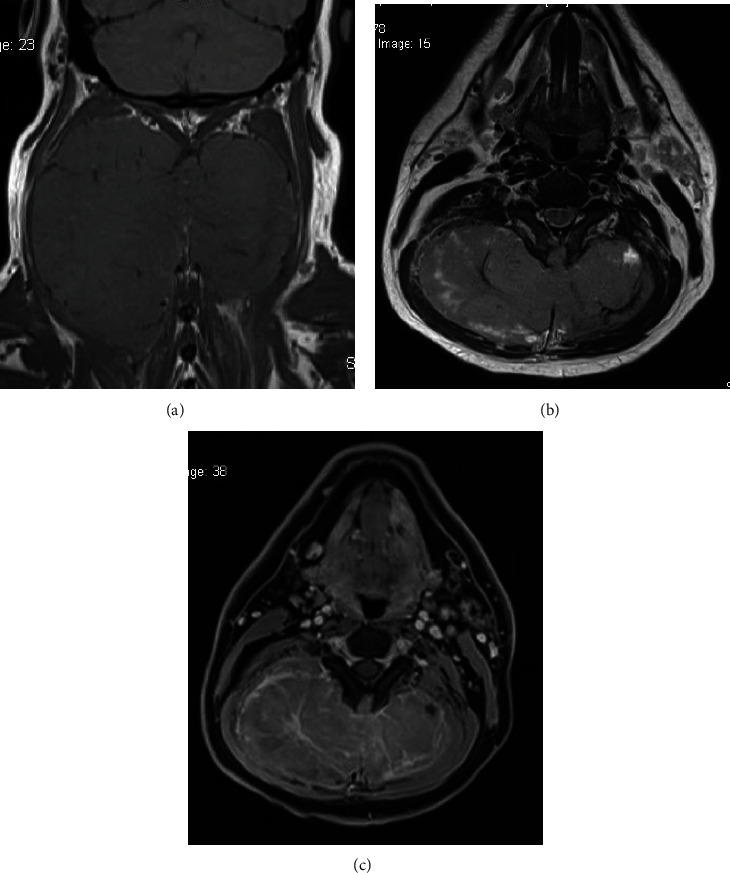
Cervical MRI: (a) coronal T1-weighted MRI and (b) axial T2-weighted MRI imaging of the cervical spine showing a posterior neck bilobed soft tissue mass showing heterogenous intermediate signal on T1 & T2 (c) axial slice of a preoperative T1 postgadolinium contrast showing patchy and reticular post contrast enhancement. MRI, magnetic resonance imaging.

A 42-year-old male presented with a one-year history of a painless progressively enlarging mass in the posterior aspect of his neck. Examination showed a large bilateral nape of neck swelling measuring approximately 15 × 15 cm as one piece, firm in consistency and with smooth margins (Figure 1). Laboratory findings were within normal limits. MRI neck done on admission findings of a posterior neck inter-/intramuscular bilobed soft tissue mass measuring 13.8 × 12.5 × 7 mm larger on the right side, the mass shows heterogeneous intermediate signal in T1 and T2 with patchy and reticular post contrast enhancement with internal areas of degeneration and area of diffusion retraction. It was not suppressed on FAT sat images. It also shows c2/3/4/5 interspinous extension with elevated marrow signal of their spinous processes and relative central canal narrowing (Figure 2).

**Figure 3:**
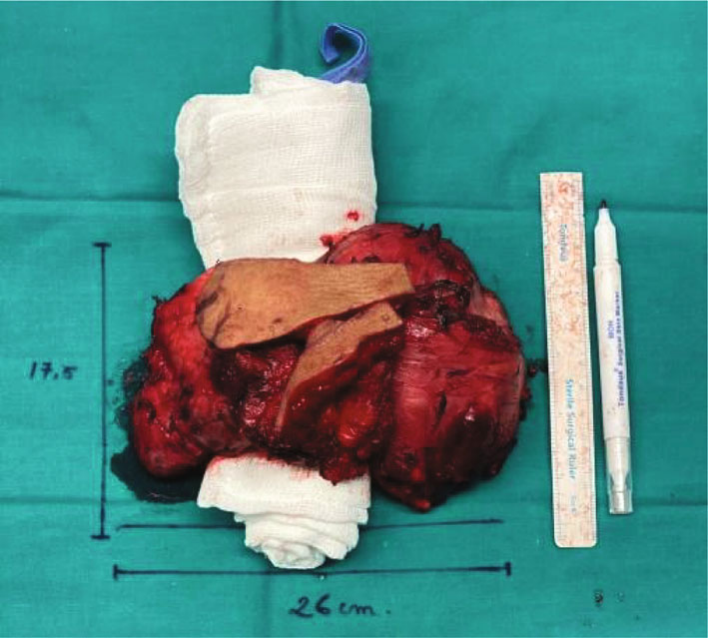
The mass post excision.

**Figure 4:**
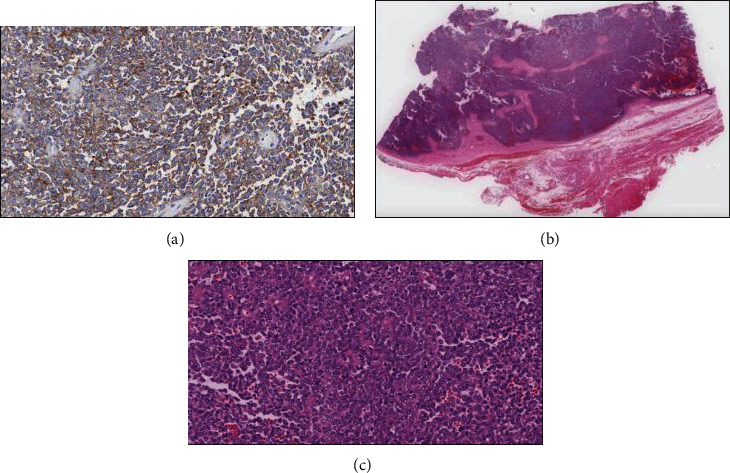
(a) Immunohistochemically, the tumor cells show diffuse membranous expression for CD99. (b) Scanning magnification (H&E stain) shows a malignant small round blue cell tumor arising from surrounding soft tissue. (c) At lower magnification (H&E stain 10x), a highly cellular, high-grade sarcoma with a dense solid- to sheet-like distribution of small round blue cells is observed histologically.

Further investigations included PET CT which revealed a hypermetabolic soft tissue mass at the posterior aspect of the neck measuring 144 × 124 × 75 mm “dumbbell-shaped soft tissue mass” and bilateral deep cervical lymph nodes at multiple levels with insignificant FDG uptake. Core biopsy revealed to have blue cell tumor most likely Ewing's sarcoma. The patient thereafter received 6 cycles of preoperative neoadjuvant chemotherapy: Vincristine, Ifosfamide, Doxoroubicin, and Etoposide (VIDE) regimen 3 doses per cycle. He did not receive any radiotherapy. Post cycles, the mass regressed in size and became softer in consistency. A postchemotherapy restaging PET CT showed interval decrease in metabolic activity of the preexisting hypermetabolic soft tissue mass in the posterior neck and the size measuring 139 × 116 × 75 mmand few bilateral cervical lymph nodes persistently seen with no significant FDG uptake. The CT cervical spine done preoperatively showed a huge bilobed intermuscular soft tissue mass occupying the whole posterior compartment of the neck from the c2 level down to the c6 level with significant stretching of both superficial and deep posterior neck muscles. The mass is semihomogenous in density with no evidence of central breakdown or calcifications. A very thin layer of fat is seen surrounding the mass all around separating it from the surrounding muscles. However, it appears very adherent to the deep posterior neck muscle layer (erector spinae muscle). The lesion invades the interspinous spaces between c2, c3, and c4 spinous processes minimally eroding them, but there is no evidence of deep invasion into the spinal canal or involving the posterior neural arches at other levels. On the 10th of January 2021, the patient underwent a major surgical excision of the mass under the care of surgical oncology, spine surgery, and plastic reconstruction surgery at our institution. The mass was massive and invading the outer surface of the spinous process; it was shaved off with the attached muscles (Figure 3). His immediate post operation period was uneventful. The patient had a Miami J collar fixed for a minimum of 2-3 months to assist in neck stability. The histopathology results after surgery confirmed the diagnosis of Ewing's sarcoma (CD99 positive) (Figure 4).

## 3. Discussion

ES and EES previously considered separate entities have been reclassified into the Ewing sarcoma family of tumors (ESFT) which includes in its umbrella Ewing's sarcoma of bone, extraskeletal Ewing sarcoma (EES), Askin tumors of the chest wall, and primitive neuroectodermal tumors of bone or soft tissues [7, 8]. These tumors are classified together due to them all having a similar molecular pattern, cytogenetic code, and response to Ewing-based chemotherapy regimens. The cytogenetic pattern present in ESFT is a reciprocal translocation between the EWS gene on chromosome 22 and the FLI-1 gene on chromosome 11, t(11;22) (q24;q12) which occurs in more than 80 to 90% of cases of ESFTs [6–8]. The expression of the CD99 glycoprotein is nearly universally present in ESFT which is encoded in the MIC2 gene [6].

EES tends to present more commonly in adult populations when compared to Ewing sarcoma which rarely presents past the third decade of life [7, 9]. Patients usually present with a painless mass or symptoms related to the tumor site. In regard to our reported case, patients with an EES in the head or neck region present with neck pain, limited cervical motion, local neurological deficits, or radiculopathy.

Imaging options used in the diagnosis of ESFT tumor status, local resectability, and distant metastases are achieved via the use of MRI, CT, and PET/CT imaging modalities [7, 10]. PET/CT is superior for the diagnosis of metastatic disease and the surveillance for recurrence of local and distant metastatic disease [7, 11]. Metastatic disease tends to be present in 20% of patients on diagnosis of ESFT [12, 13]. It is essential to diagnose metastatic disease early and treat aggressively, as 5-year survival of lung or bone metastases decreases to 40% and 20%, respectively, when compared to nonmetastatic disease [7, 11–13].

There are no specific radiological features for EES; however, they are usually described as well circumscribed noncalcific soft tissue tumors, which can demonstrate internal heterogeneity with areas of low attenuation secondary to necrosis or hyperattenuation secondary to hemorrhage on computed tomographic examination. Lymphadenopathy due to metastasis is very rare and is only reported in 0-12% of cases [14]. Definitive diagnosis however can only be made by histopathological studies.

EES is considered to be a poor prognostic factor to survival when compared to skeletal Ewing sarcoma, due to its propensity for invasion of local adjacent organs and metastasizing to the lungs [4, 5]. According to a recent review from Carballo Cuello et al., the survival for EES remains dismal: their one-, two-, and five-year progression-free survivals are 36.4%, 36.4%, and 12.1%, respectively, whereas the one-, two-, and five-year overall survival rates are 72.7%, 62.3%, and 46.8%, respectively [15]. Treatment of ES and EES usually involves the use of neoadjuvant chemo- or radiotherapy and en bloc resection of the localized tumor [4, 14].

Upon diagnosis of ESFT, the initial treatment involves induction chemotherapy with Vincristine, Ifosfamide, Doxoroubicin, and Etoposide (VIDE) regimen for 6 cycles at 2–4-week intervals [14, 15]. After induction chemotherapy, patients undergo local therapy with either surgical resection if the tumor can be resected or radiotherapy if it cannot, followed by consolidation therapy with Vincristine, Actinomycin D, and Cyclophosphamide (VAC) or Vincristine, Actinomycin D, and Ifosfamide (VAI) regimen [15, 16]. High-risk patients will undergo high-dose chemotherapy with Busulphan and Melphalan followed by stem cell rescue options [14, 16]. Risk stratification of patients is based upon tumor size, minimal or no response to preoperative chemotherapy, disease relapse, and presence of metastases upon diagnosis [16]. The use of chemotherapy regimens and local treatment options has increased 5-year survival of nonmetastatic ESFT from around 10% in the 1960s to nearly 60% [16–18].

Dumbbell tumors are defined as tumors with both intraspinal and anterior paraspinal components that communicate via an intravertebral foramen [18]. About 14-20% of spinal tumors are dumbbell tumors in which most are neurogenic tumors in origin [9, 19]. Dumbbell tumors usually tend to be benign tumors such as schwannomas more often than malignant tumors such as peripheral nerve sheath tumors. In addition schwannomas are more likely to be dumbbell than intraspinal [20]. It is even more rare for EES to be characterized as a dumbbell tumor [21].

Due to the scant and limited amount of literature available on metastatic spinal dumbbell tumors (SDT) and especially that on EES presenting as a SDT, we believe our case is rare in its presentation.
